# Physiological implications of NTBI uptake by T lymphocytes

**DOI:** 10.3389/fphar.2014.00024

**Published:** 2014-02-26

**Authors:** Jorge P. Pinto, João Arezes, Vera Dias, Susana Oliveira, Inês Vieira, Mónica Costa, Matthijn Vos, Anna Carlsson, Yuri Rikers, Maria Rangel, Graça Porto

**Affiliations:** ^1^Molecular and Cellular Biology Division, Basic and Clinical Research on Iron Biology, Instituto de Biologia Molecular e Celular, Universidade do PortoPorto, Portugal; ^2^Faculdade de Medicina, Universidade do PortoPorto, Portugal; ^3^Europe NanoPort, FEIEindhoven, Netherlands; ^4^Chemistry Department, REQUIMTE, Instituto de Ciências Biomédicas Abel Salazar, Universidade do PortoPorto, Portugal; ^5^Clinical Hematology, CHP-HSA - Santo António General HospitalPorto, Portugal; ^6^Molecular Immunology and Pathology, Instituto de Ciências Biomédicas Abel Salazar, Universidade do PortoPorto, Portugal

**Keywords:** iron, homeostasis, NTBI, lymphocytes, hemochromatosis

## Abstract

In iron overload disorders a significant fraction of the total iron circulates in the plasma as low molecular weight complexes not bound to transferrin, known as non-transferrin-bound iron (NTBI). By catalyzing the formation of free radicals, NTBI accumulation results in oxidative stress and cellular damage, being a major cause of organ toxicity. NTBI is rapidly and preferentially cleared from circulation by the liver and the myocardium, the main disease targets in iron overload conditions. We have recently demonstrated that human peripheral blood T lymphocytes take up NTBI *in vitro*, with a pattern that resembles that of hepatocytes. Since T lymphocytes constitute a numerically important component of the circulating cell pool, these findings support a putative role for this cell type in the systemic protection against iron toxicity. Here we tested the hypothesis that the circulating peripheral blood T lymphocyte pool constitutes an important storage compartment for NTBI and is thus a modifier of NTBI deposition in target organs. First we show that NTBI uptake by human T lymphocytes increases the expression of the iron-storage protein ferritin and of the iron exporter ferroportin *via* an IRE-dependent mechanism. NTBI retention by T lymphocytes is shown to be critically controlled by the hepcidin-mediated modulation of ferroportin both *in vitro* and *in vivo*. Finally, the protective effect of T lymphocytes was tested by analyzing the patterns of iron accumulation in the T lymphocyte-deficient mouse model *Foxn1^nu^* before and after reconstitution with T lymphocytes by adoptive transfer. The results confirmed a significant increase of liver and pancreas iron accumulation in T lymphocyte-deficient mice. NTBI accumulation in the liver and spleen was prevented by reconstitution with syngeneic T lymphocytes. Altogether, our results demonstrate that T lymphocytes are important components of a circulating “NTBI storage compartment” and show its physiological relevance as a modifier of tissue iron overload.

## Introduction

Iron, the most abundant transition metal in mammalian systems, is essential for various vital metabolic processes. In physiologic conditions iron circulates in the plasma bound to transferrin, the main iron transporter protein, and this constitutes the major iron source for iron-avid processes, such as erythropoiesis (Hentze et al., 2010).Circulating iron which is not bound to transferrin, heme or ferritin (here designated as non-transferrin-bound iron—NTBI) becomes relevant in iron overload disorders, appearing in plasma even before transferrin becomes fully saturated (Breuer et al., 2000; Esposito et al., 2003).

NTBI is the main source of iron for storage in the liver (Zimelman et al., 1977; Brissot et al., 1985). However, in contrast to transferrin-bound-iron, NTBI is potentially toxic, causing cellular damage, not only of the plasma membrane but also of various intracellular organelles, due to its involvement in the formation of reactive oxygen species (reviewed in Brissot et al., 2012). The uptake of NTBI by hepatocytes is thus viewed as a clearance mechanism of potentially toxic circulating iron that could otherwise cause damage to other cell types. Not surprisingly, hepatocytes are the first target of iron toxicity in situations of severe iron overload such as in HFE-Hereditary Hemochromatosis (HFE-HH) or transfusion-dependent beta-thalassemia. When the clearance capacity of liver is exceeded, other organs, namely pancreas, heart or hypophysis, are also affected by NTBI uptake and accumulation, leading to the full-blown clinical picture of severe iron overload with liver cirrhosis, diabetes, cardiomyopathy, and hypogonadotrophic hypogonadism (Pietrangelo, 2004). Therefore an effective removal of circulating NTBI is a major goal in the management of iron overload disorders.

We have recently demonstrated that T lymphocytes are capable of taking up and accumulating the same NTBI species as hepatocytes (Arezes et al., 2013). Together with previous evidence showing that T lymphocytes synthesize ferritin in greater amounts than non-T lymphoid cells (Dörner et al., 1980) these results support the hypothesis that T lymphocytes may have an important role in iron handling in the blood circulation, where they could act as natural NTBI buffers and constitute a barrier to protect other tissues from iron-mediated toxicity. In order to test this hypothesis we analyze here the capacity of T lymphocytes to store/export NTBI and assess the physiological implications of NTBI storage by T lymphocytes as a modifier of tissue iron load.

## Methods

### Isolation of human peripheral blood cells

Peripheral Blood Mononuclear cells (PBMCs) were obtained from apparently healthy volunteer blood donors, randomly recruited at Santo António Hospital Blood Bank (Porto, Portugal) who gave their consent to participate in this study, approved by the Santo António Hospital Ethical Committee. Cells were isolated by gradient centrifugation over Lymphoprep (Nycomed). After lysis of erythrocytes, cells were resuspended in RPMI (GibcoBRL) supplemented with 10% fetal calf serum (FCS; GibcoBRL) and plated. CD3^+^, CD4^+^, and CD8^+^ cells were purified from PBMCs using magnetic-activation cell sorting (MACS), after incubation with specific microbead-conjugated antibodies (Miltenyi Biotec), according to manufacturer's instructions.

### NTBI uptake

Uptake of non-transferrin-bound iron (NTBI) was assessed using ^55^Fe-citrate (Grootveld et al., 1989). ^55^Fe-citrate stock solutions were prepared by mixing ^55^FeCl_3_ (5–10 mCi, in 0.1 M HCl; Amersham) with unlabeled trisodium citrate, for a final citrate concentration of 100 μM. The pH was maintained at 7.4 and solutions were allowed to rest for 20 min before being diluted 33-fold in uptake medium and added to cells. Specific activity in the uptake medium was approximately 30 counts· min^−1^· pmol^−1^ Fe. All Fe:citrate solutions were freshly prepared before use and discarded after each experiment. To prevent competition of T lymphocyte-secreted transferrin for citrate-bound iron, unless otherwise indicated cells were depleted of intracellular transferrin by incubation for 1 h in serum-free RPMI. Since the estimated time of recycling of transferrin molecules in lymphocytes is approximately 30 min (Holtzman, 1939), 1 h should suffice for all endogenous transferrin to be secreted and, due to the absence of extracellular iron, be prevented from being endocytosed again. Cells were then washed and incubated with RPMI + 20% FCS + 5 μM ^55^Fe-citrate (as 5 μM ^55^FeCl_3_+ 100 μM citric acid), at 37°C. Given the 5 μM is the typical NTBI concentration reported in sera from *thalassemia major* patients (Evans et al., 2008) and 100 μM citric acid falls within the interval of citrate concentrations normally present in human blood plasma (Lentner, 1984). In some experiments the iron chelator desferrioxamine (DFO; Sigma) was added to the medium providing a final concentration of 5 μM. The pH of the incubation medium was maintained at 7.4. After incubation, cells were washed 3× with ice-cold washing buffer (20 μM DFO, in PBS, pH 7.4), lysed with a 0.1% NaOH, 0.1% Triton X-100 solution and intracellular Fe was measured in a MicroBeta Trilux β-counter (Perkin Elmer), for 1 min. No significant impact of iron treatments in cell viability was observed, using trypan blue exclusion and maintenance of proliferative potential following activation with anti-human anti-CD3 and anti-human anti-CD28 for CD3^+^, CD4^+^, and CD8^+^ T lymphocytes (Arezes et al., 2013).

### Iron export

T lymphocytes were depleted of transferrin, as described above, and incubated, unless otherwise stated, in RPMI + 5 μM Fe-citrate (or 5 μM ^55^Fe-citrate) + 20% FCS for 2 h. Cells were washed 2× with washing buffer and incubated for different time-periods in RPMI + 20% FCS + 5 μM DFO (for short-term experiments), to prevent re-uptake of exported iron, or in RPMI + 20% FCS (for long-term experiments). At each time-point, the supernatants were collected and Fe was quantified as described in *NTBI uptake*.

To analyze the effect of hepcidin in iron export, CD4^+^ and CD8^+^ T lymphocytes were iron-loaded with 5 μM Fe-citrate (or 5 μM ^55^Fe-citrate), as described above, washed and incubated in export medium (RPMI + 20% FCS) supplemented with 100 or 600 ng/ml of human synthetic hepcidin (Peptides International), or with an equal volume of PBS (Mock), for up to 72 h. FPN levels were analyzed by western blot analysis (see below) after 24 h of incubation and intracellular ^55^ Fe levels were quantified after up to 72 h of incubation.

### Assessment of labile iron pool

CD4^+^ and CD8^+^ T lymphocytes were loaded with 0.25 μM of calcein acetoxymethyl ester dye (Molecular Probes) at 37°C for 10 min, and rinsed twice with PBS to remove unincorporated dye. These conditions were empirically determined by us to be the most suitable for detection of iron-induced changes in calcein fluorescence in PBMCs (Pinto et al., unpublished data). Cells were then incubated with 5 μM of Fe-citrate (5:100) + 20% FCS or with 5 μM of Na-citrate + 20% FCS, for up to 180 min, and the fluorescence intensity was measured at defined time points in a fluorescent plate reader, using the conditions λ_exc_ = 488 nm and λ_em_ = 517 nm. In some experiments, the membrane permeable iron chelator salicylaldehyde isonicotinoyl hydrazone (SIH, kindly provided by Dr. Prem Ponka, McGill University, Canada) was added to the cells (10 μ M final concentration) for 30 min following incubation with iron.

### Western blot analysis

Cellular extracts were isolated by the carbonate fractioning procedure, using a protocol adapted from Fujiki et al. (1982). Briefly, harvested CD3^+^ lymphocytes were resuspended in 120 mM sodium carbonate supplemented with 1× Complete EDTA-free protease inhibitor cocktail (Roche Diagnostics) and incubated on ice for 30 min. An aliquot of this suspension was saved as total fraction. The suspensions were transferred to polycarbonate tubes and centrifuged at 38,000 rpm for 45 min, at 4°C, using a Beckman 70.1 Ti rotor. The supernatants (corresponding to the cytoplasmic fractions) were decanted and the membrane pellets were directly dissolved, at 65°C, in 2× Laemmli buffer, with occasional vortexing and sonication. Both total and soluble extracts were subjected to trichloroacetic acid precipitation and processed for SDS-PAGE.

Cell extracts were submitted to SDS-PAGE and immunoblotted for ferritin (1:1000 rabbit anti-ferritin antibody; Abcam) and/or ferroportin (1:100 rabbit anti-ferroportin antibody; Novus Biologicals). To control for the purity of cell fractions, some of the extracts were hybridized with an anti-alpha 1 Na/K ATPase antibody (1:2000; Abcam). Immuno-reactive proteins were detected with Super Signal West Dura (Pierce). For control of protein loading, membranes were stripped and re-hybridized with 2 μg/ml of a mouse anti-human β-actin or goat polyclonal anti-β-actin antibodies (both from Abcam).

### Intracellular Fe detection by energy dispersive X-ray analysis

Lymphocytes were separated from monocytes in PBMCs by allowing monocytes to adhere to the substrate for 1.5 h and collecting the supernatant (lymphocyte-fraction). Cells were depleted of transferrin and incubated with 5 μM Fe-citrate (5:100) for 24 h. Cells were pelleted, fixed in 2.1% glutaraldehyde/2% formaldehyde, washed with 0.1 M phosphate buffer and fixed for 1 h with 1% OsO_4_, at 4°C. After dehydration, the samples were embedded in Epon 812 and ultrathin sections obtained. For high resolution fast elemental mapping of iron-loaded lymphocytes, a FEI Tecnai Osiris transmission electron microscope with ChemiSTEM technology was used. Images were recorded using a Fischione High Angle Annular Dark Field (HAADF) detector. A FEI super-X EDX detector was used, which provides a solid collection angle of 0.9 sterads fully integrated with the A-TWIN objective lens. The elemental maps were recorded with a 20 μs dwell time per pixel (spectra) with a 1K × 1K frame size (approximately 20 s scan time per total frame). As plastic sections are susceptible to shrinkage and beam damage, care was taken not to expose the sample outside of the acquisition period by blanking the beam when no image was acquired. Before scanning, samples were pre-eradiated in TEM mode at low magnification for 30 min to reach a stable state with respect to shrinkage. During and after the period of acquisition, the scanning area was assessed with respect to the contrast of the image, which was taken as a measurement of stability. No additional shrinkage or carbon deposit occurred during the scanning period as the contrast of the image did not change after each consecutive scan. We assumed therefore that, after pre-eradiation, the sample was in a stable state and no additional mass loss occurred, nor contamination deposited on the sample during acquisition, showing that both the beam current was acceptable for the imaging and the column contamination was negligible. The spectra were processed using Bruker Esprit software and the elemental map of Fe was calculated. The Energy Dispersive X-ray (EDX) maps were processed with an opening filter followed by a Gaussian smoothing.

### Plasmid construction and luciferase reporter assay

The promoter region of *FPN1* spanning from 845 bp 5′ to the ATG codon to 50 bp after *FPN1* transcription initiation site was amplified and ligated into the pGL4 luciferase reporter vector (Promega). pGL4 has an SV40 promoter, driving the transcription of a luciferase chimeric mRNA which includes the *FPN1* 5′UTR. The *FPN1* 5′CAGUG IRE sequence, which is involved in the formation of the terminal –AGU- loop (Hentze and Kuhn, 1996) was mutagenized into CCCCG with the Quickchange® II site direct mutagenesis kit (Stratagene), according to the manufacturer's protocol. The luciferase chimeric constructs containing the wt and mutated *FPN1* IREs were transfected into CD4^+^ and CD8^+^ cells using AMAXA nucleofection, as previously described (Pinto et al., 2010). The day after nucleofection, cells were incubated for 2 h with 5 μM of Fe-citrate (5:100), 5 μM of Fe-citrate (5:100) + 5 μM of DFO or with RPMI, and lysed. The luciferase activity was measured with the Dual-Luciferase Reporter Assay System (Promega), according to the manufacturer's instruction. The luciferase activity was normalized with renilla luciferase activity, used as transfection control. To control for changes in promoter-driven transcription, luciferase mRNA levels were assessed in parallel, with no significant changes observed between each experimental condition (data not shown).

### Gene expression

Total RNA was extracted using the RNeasy Plus Mini kit (Qiagen), with on-column DNA digestion (Qiagen). cDNA was synthesized using the Superscript First-Strand Kit (Invitrogen) and qRT-PCR was performed in an iCycler iQ5 PCR detection system (Bio-Rad), using specific primers. Glyceraldehyde-3-phosphate dehydrogenase (*GAPDH*) and 18S rRNA mRNA expression were used as internal controls. Since no significant differences were observed between the two controls, all qRT-PCR results displayed were normalized for *GAPDH* mRNA expression. Melting curve experiments previously established that the signal for each amplicon was specific and not derived from primer dimers. For every gene a series of four serial dilutions was used during optimization of the procedure. Relative expression levels were calculated as 2^−ΔΔ*CT*^ [ΔΔCT = (Gene of interest CT − *GAPDH* CT)_sample_ − (Gene of interest CT − *GAPDH* CT)_reference_]. All experiments involving qRT-PCR were performed at least in triplicate, with two to three replicates each.

### Experimental procedures with mouse models

All mice models used were 7–8 week old females, maintained at the IBMC's Animal Care Facility. C57BL/6 mice were supplied by Charles River or Jackson Laboratory. *Foxn1^nu^* and *Foxn1*^+/−^ were purchased from the Jackson Laboratory. All experimental procedures were approved by and performed according to the guidelines of the IBMC's Animal Care Committee.

For the analysis of the effect of exogenous hepcidin on ferroportin expression mice (*n* = 6) were maintained on an iron-sufficient diet (35 mg/kg chow; Harlan Laboratories) from weaning and intravenously injected daily with mouse hepcidin (Peptides International; 50 μg/mouse in 0.3 ml PBS) for 3 consecutive days. The control group (*n* = 6) was injected with PBS only. Animals were killed 3 days after the first dose injection and peripheral blood CD3^+^ cells isolated with MACS, as described above.

To study the effect of endogenous hepcidin on ferroportin expression mice (*n* = 12/experimental condition) were maintained on the iron-sufficient diet from weaning or on iron-rich diet (iron-sufficient diet enriched with 2.5% iron carbonyl—Sigma) for 2 weeks. Mice were then intravenously injected each 24 h with 100 μl of 50 μM ^55^Fe-citrate (50:100), to achieve a systemic, traceable, ^55^Fe-citrate concentration of 5 μM. Two days after the first ^55^Fe injection mice were sacrificed and livers and peripheral blood CD3^+^ cells were collected. *Hamp1* mRNA expression was quantified in the livers by qRT-PCR and Fpn1 expression and intracellular ^55^Fe accumulation quantified in CD3^+^ T lymphocytes, as described above.

The effect of each diet on blood iron-related parameters was assessed in 4 animals/experimental condition by analyzing levels of serum iron, total iron binding capacity, and resulting transferrin saturation by standard routine procedures at the Clinical Chemistry Laboratory of Santo António Hospital (Porto).

For the study of the modifier role of T lymphocytes in NTBI accumulation *Foxn1^nu^* and *Foxn1*^+/−^ mice were fed the iron-rich diet for 2 weeks and intravenously injected, every 2 days, with 100 μl of a 50 μM ^55^Fe-citrate (50:100) solution, during 7 days. The animals were killed 8 days after the first dose injection and the livers and spleen collected. Incorporation of ^55^Fe was quantified following the same procedure described in *NTBI uptake*.

For T lymphocyte reconstitution of *Foxn1^nu^* mice, CD3^+^ lymphocytes were isolated from the spleens of *Foxn1^+/−^* animals (on iron-sufficient diet) using MACS, resuspended in 100 μl of endotoxin-free PBS and intravenously injected via the lateral tail vein (5 × 10^6^ or 15 × 10^6^ T lymphocytes/injection) on *Foxn1^nu^* mice that had been fed the iron-sufficient diet from weaning or the iron-rich diet for 2 weeks. Control (Mock) animals were injected with an equal volume of PBS. The purity of the T lymphocyte fraction was verified by FACS, using an FITC-labeled anti-mouse CD3 antibody (BD Pharmingen) and confirmed to be >93%. Immediately following T lymphocyte transfer mice on iron-rich diets were injected with 100 μl of a 50 μM ^55^Fe-citrate (50:100) solution, which was repeated 24 h later. Forty-eight hours after T lymphocyte transfer all mice were sacrificed and the levels of ^55^Fe in the liver and spleen quantified. For quantification of hemosiderin deposition, livers were fixed in 4% formaldehyde, embedded in paraffin, mounted onto slides, and stained with Prussian blue and hematoxylin-eosin counterstain, using standard procedures. Hemosiderin was detected using the Perls' Prussian Blue method.

### Statistical analysis

The results are expressed as mean values ± 1 standard deviation (SD). Statistical differences between means were calculated using the Student's unpaired *t*-test. For experiments involving multiple comparisons, One-Way or Two-Way analysis of variance (ANOVA) was used. When significant differences were detected, the data were re-analyzed using the Fisher's least significance difference test. Statistical significance was set at *P* < 0.05. All statistical analyses were performed using STATGRAPHICS Centurion XV (Statpoint Technologies).

## Results

### NTBI accumulation by human peripheral blood T lymphocytes

**Figure 1:**
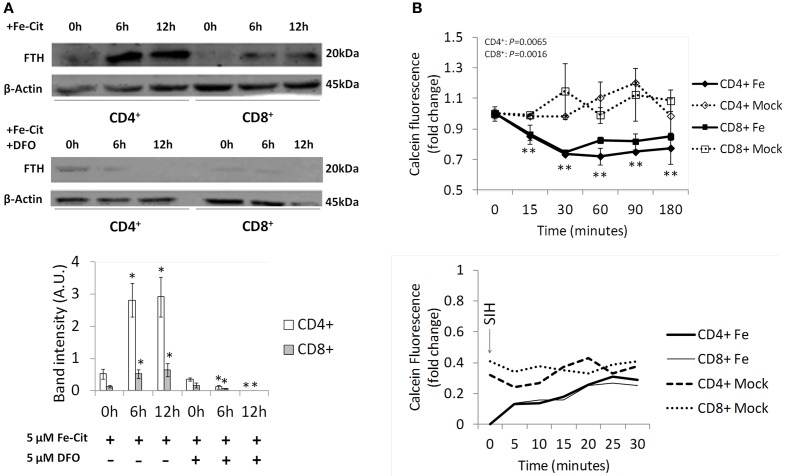
**NTBI storage by T lymphocytes. (A)** Regulation of Ferritin H expression by NTBI in T lymphocyte populations. Western blot quantification of Ferritin H (FTH) expression in CD4^+^ and CD8^+^ cells incubated with 5 μM of Fe-citrate (upper panel) or Fe-citrate + 5 μM DFO (middle panel) for 0–12 h. β-actin expression was used as loading control. Images are representative of three experiments without significant inter-experimental variation. Bottom panel: Scanning densitometry of FTH Western blot. Data are normalized for β-actin levels and are expressed as arbitrary units (AU). ^*^P < 0.05 and ^**^P < 0.005 (One-Way ANOVA) between relative FTH levels at 0 h and posterior time-points, for each population and experimental condition. **(B)** Regulation of the Labile Iron Pool by NTBI in T lymphocyte populations. Upper panel: intracellular calcein fluorescence in CD4^+^ and CD8^+^ T lymphocytes incubated with 5 μM of Fe-citrate or 5 μM of Na-citrate (Mock). Each point represents the average (*n* = 3) ± 1 SD. ^**^P < 0.005 (One-Way ANOVA) between fluorescence levels at 0 h and posterior time-points, for each population and experimental condition. Bottom panel: following incubation with 5 μM of Fe-citrate for 180 min, 10 μM of SIH were added to cells (arrow) and changes in calcein fluorescence monitored for 30 min. Data from a representative experiment (*n* = 3), with minimum inter-experimental variation.

To test the ability of T lymphocytes to store iron acquired as NTBI, CD4^+^, and CD8^+^ T lymphocytes were incubated with 5 μM of Fe-citrate for 0–12 h and the expression of the iron storage protein Ferritin H (FTH) quantified by Western blot analysis. We observed that both T lymphocyte populations increase FTH levels in response to Fe-citrate, an effect abrogated by the supplementation of the culture medium with 5 μM desferrioxamine (DFO) (Figure 1A). The changes in FTH expression are in agreement with our previous results showing that a plateau in intracellular iron is reached earlier than 6 h in the presence of a constant NTBI concentration (Arezes et al., 2013) and suggest the capacity of the cells to store a fraction of the intracellular iron in the Ferritin core.

The fraction of iron that is not included in the storage compartment should be associated with proteins other than ferritin (*functional iron*) or enter the *transit iron* pool, also known as Labile Iron Pool (LIP). Using the calcein assay, which is based on the quenching of the fluorescence of the permeant calcein acetoxymethyl ester by iron, we assessed the contribution of NTBI to the LIP in T lymphocytes. We observed a significant time-dependent decrease in the fluorescence of intracellular calcein exclusively for T lymphocyte populations exposed to 5 μM of Fe-citrate, indicating an increase in intracellular labile iron (Figure 1B). The difference between iron-loaded and control cells reaches its maximum values after 30 min of incubation, suggesting that an equilibrium is reached at this point between the formation of new labile iron and incorporation of iron in the storage pool + iron export. Validation of the specific effect of iron in the fluorescence quenching of calcein was obtained by recovering fluorescence exclusively on iron-treated cells upon incubation with the membrane-permeable iron chelator SIH.

**Figure 2:**
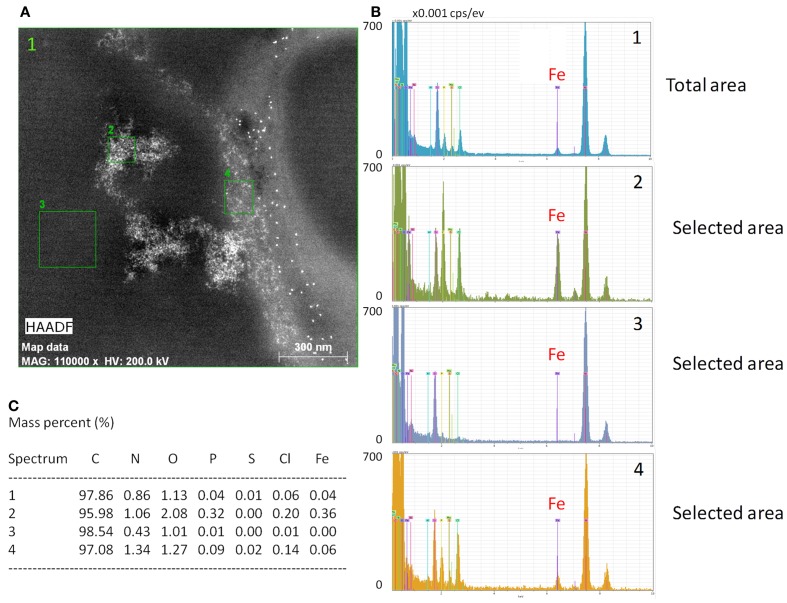
**Intracellular iron detection with EDX. (A)** Energy dispersive X-ray analysis of a representative image showing intracellular cytoplasmic iron in a CD3^+^ cell. White represents iron. Numbered areas analyzed for elemental composition; “1” represents the total area depicted. **(B)** Elemental composition of the four areas depicted in **(A)** with the peak corresponding to iron highlighted (Fe). Peak height is proportional to the respective element's abundance. Values are depicted in counts per pix per second per ev (cps/ev). **(C)** Relative (%) elemental composition of regions signaled in **(A)**; C = carbon, N = nitrogen, O = oxygen, P = phosphorous, S = sulfur, Cl = chlorine, Fe = iron.

To further characterize intracellular iron accumulation in T lymphocytes we used EDX analysis to generate maps of intracellular iron distribution in ultrathin sections of cells incubated with 5 μM Fe-citrate (5:100) for 24 h. Using this approach we confirmed that T lymphocytes accumulate iron from Fe-citrate and showed that intracellular iron shows distinct distribution patterns, ranging from homogeneously-sized particles with an area of approximately 20 nm^2^ to large aggregates with a more amorphous appearance, some of which >1 μm in diameter (Figure 2A). A more detailed analysis of the iron-rich structures showed that their iron content is highly variable (Figures 2B,C), revealing also a consistent enrichment in nitrogen, oxygen, phosphorous, and chlorine (Figure 2C), which suggests the association of intracellular iron with other compounds, that could include phosphate and proteins.

### NTBI export by human peripheral blood T lymphocytes

#### Iron export

**Figure 3:**
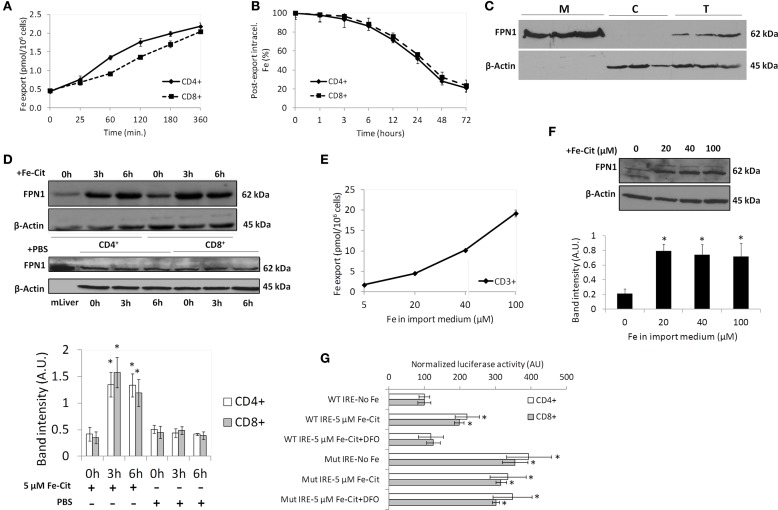
**Iron export by T lymphocytes**. **(A)** Time-dependent iron export by T lymphocytes. Each point is a mean value (*n* = 3) ± 1 SD. **(B)** Impact of iron export in intracellular Fe levels. Each point is a mean value ± 1 SD of two experiments each with three replicates. **(C)** Ferroportin (FPN1) expression in CD3^+^ T lymphocytes. Membrane (M), cytoplasmic (C), and total (T) extracts of CD3^+^ cells were blotted and membranes incubated with anti-FPN1 and anti-β-actin antibodies. Results obtained with CD3^+^ cells from three different blood donors are presented. **(D)** Western blot quantification of FPN1 expression in CD4^+^ and CD8^+^ T lymphocytes incubated with 5 μM of Fe-citrate (upper panel) or PBS (middle panel) for 0–6 h. β-actin expression was used as loading control. Images are representative of four experiments without significant inter-experimental variation. Bottom panel: Scanning densitometry of FPN1 Western blots. Data are normalized for β-actin levels and are expressed as arbitrary units (AU); ^*^P < 0.05 (One-Way ANOVA) between relative FPN1 levels at 0 h and posterior time-points, for each population and experimental condition. **(E)** Dose-dependent iron export by T lymphocytes. Each point is the mean of two experiments with two replicates each. **(F)** Western blot quantification of FPN1 expression in T lymphocytes incubated with different Fe-citrate concentrations. β-actin expression was used as loading control. Images are representative of three experiments without significant inter-experimental variation. Bottom panel: Scanning densitometry of FPN1 Western blot. Data are normalized for β-actin levels and are expressed as arbitrary units (AU). ^*^P < 0.05 (One-Way ANOVA) between relative FPN1 levels at 0 h and posterior time-points. **(G)** Involvement of the IRE/IRP system in the NTBI-induced modulation of FPN1 in T lymphocytes. Cells were transfected with a chimeric construct including wild-type (WT) or mutated (Mut) FPN1-IRE and incubated with Fe-citrate (Fe-Cit), Fe-Cit + DFO or RPMI (No-Fe) and the luciferase activity quantified. Each point is a mean value (*n* = 3) ± 1 SD. ^*^P < 0.05 (One-Way ANOVA) for each experimental condition relative to WT IRE-No Fe control.

In order to have a relevant role as a component of the *iron storage* compartment, T lymphocytes would need to selectively retain or, alternatively, export intracellular iron acquired as NTBI according to systemic signals. To test this hypothesis we analyzed the export of iron acquired as Fe-citrate by T lymphocytes. CD4^+^ and CD8^+^ T cells were exposed to 5 μM Fe-citrate for 2 h, a time point at which cells have reached the maximum iron content (Arezes et al., 2013), and allowed to export iron into an iron-free medium for up to 6 h. We observed that iron export by T lymphocytes follows a linear pattern, with a delay in CD8^+^ cells, which only match CD4^+^ lymphocyte's export rate after 60 min of export (Figure 3A). After 60 min in an iron-free medium, iron export by T lymphocytes corresponds to approximately 3% of intracellular levels, demonstrating a slow release of iron acquired as NTBI by these cells. This is confirmed by the quantification of intracellular ^55^Fe remaining in both T lymphocyte populations throughout time, which further shows that, after 72 h of export, T lymphocytes maintain approximately 20% of the initial iron load acquired as NTBI (Figure 3B).

#### Role of FPN1

The trans-membrane domain protein SLC40A1 (ferroportin, FPN1) is the only known cellular iron exporter (Donovan et al., 2005). Little is known about the expression of FPN1 by T lymphocytes and even less on its regulation by cellular and systemic stimuli. We observed clear expression of the protein in membrane extracts of untreated CD3^+^ cells, while it was undetectable in the cytosolic fraction (Figure 3C). The high purity of the cell fractionation was further confirmed by the clear detection of the plasma membrane marker protein alpha 1 sodium potassium ATPase in one of the membrane extracts depicted in Figure 3C, with only residual detection in the cytoplasmic extracts (Supplementary Figure 1). Expression of FPN1 increased significantly in T lymphocytes after exposure to 5 μM Fe-citrate for 3 h and are maintained at least up to 6 h of incubation (Figure 3D). No changes in FPN1 were observed in cells supplemented with PBS, confirming the role of iron in the observed FPN1 increase. Altogether, these results suggest the involvement of ferroportin in the trans-membranar transport of iron in T lymphocytes.

Although 5 μM Fe-citrate is in the range of the NTBI concentrations commonly detected in iron overload disorders, in severe cases of iron overload values as high as 30 μM have been reported (Batey et al., 1980). To test whether iron export by T lymphocytes is dependent on the extracellular NTBI concentration, cells were incubated with increasing doses of Fe-citrate (XFe:100 μM citrate) for 2 h and allowed to export iron into an iron-free medium. We observed a dose-dependent increase in iron export with increasing NTBI concentrations (Figure 3E). Interestingly, the increase in iron export cannot be accounted by augmented FPN1 levels, as no significant changes in FPN1 expression were detected between incubations with 20 and 100 μM Fe-citrate (Figure 3F). The increased iron export might thus be caused by an increase in the flux of Fe_*(II)*_ ions transported by each FPN1 molecule rather than to a augmented number of FPN1 molecules at the cell surface.

In hepatocytes and macrophages intracellular iron regulates FPN1 translation, by a mechanism involving the IRP/IRE machinery (Lymboussaki et al., 2003). To test if this mechanism is involved in the response of FPN1 to NTBI in T lymphocytes, we cloned the FPN1-5′UTR in the pGL4 vector, in which a SV40 promoter drives transcription to produce a luciferase chimeric mRNA with the FPN1-5′UTR as its 5′UTR. In CD4^+^ and CD8^+^ cells nucleofected with the FPN1/luciferase construct, the expression of luciferase increased approximately 2-fold following incubation with 5 μM Fe-citrate for 2 h (Figure 3G), in comparison with cells incubated in iron-free medium or in medium containing Fe-citrate + DFO. This result matches the increase in FPN1 observed by immuno-blot (Figure 3D). Sequential mutagenesis of the FPN1-5′-UTR previously shown to impair IRP binding to FPN1-IRE (Hentze and Kuhn, 1996) resulted in a significant increase (approximately 4-fold) of the basal FPN1 expression in the two cell populations and, importantly, abrogated the response to Fe-citrate. Together, these results confirm the involvement of the IRE/IRP system in the NTBI-mediated increase in FPN1 expression. This post-transcriptional mechanism is probably the major contributor to the FPN1 increase in response to NTBI, as no changes in *FPN1* mRNA expression were observed in response to 5 μM Fe-citrate (Supplementary Figure 2), arguing against a transcriptional regulation of FPN1 by NTBI in these cells.

#### The hepcidin-FPN1 axis

**Figure 4:**
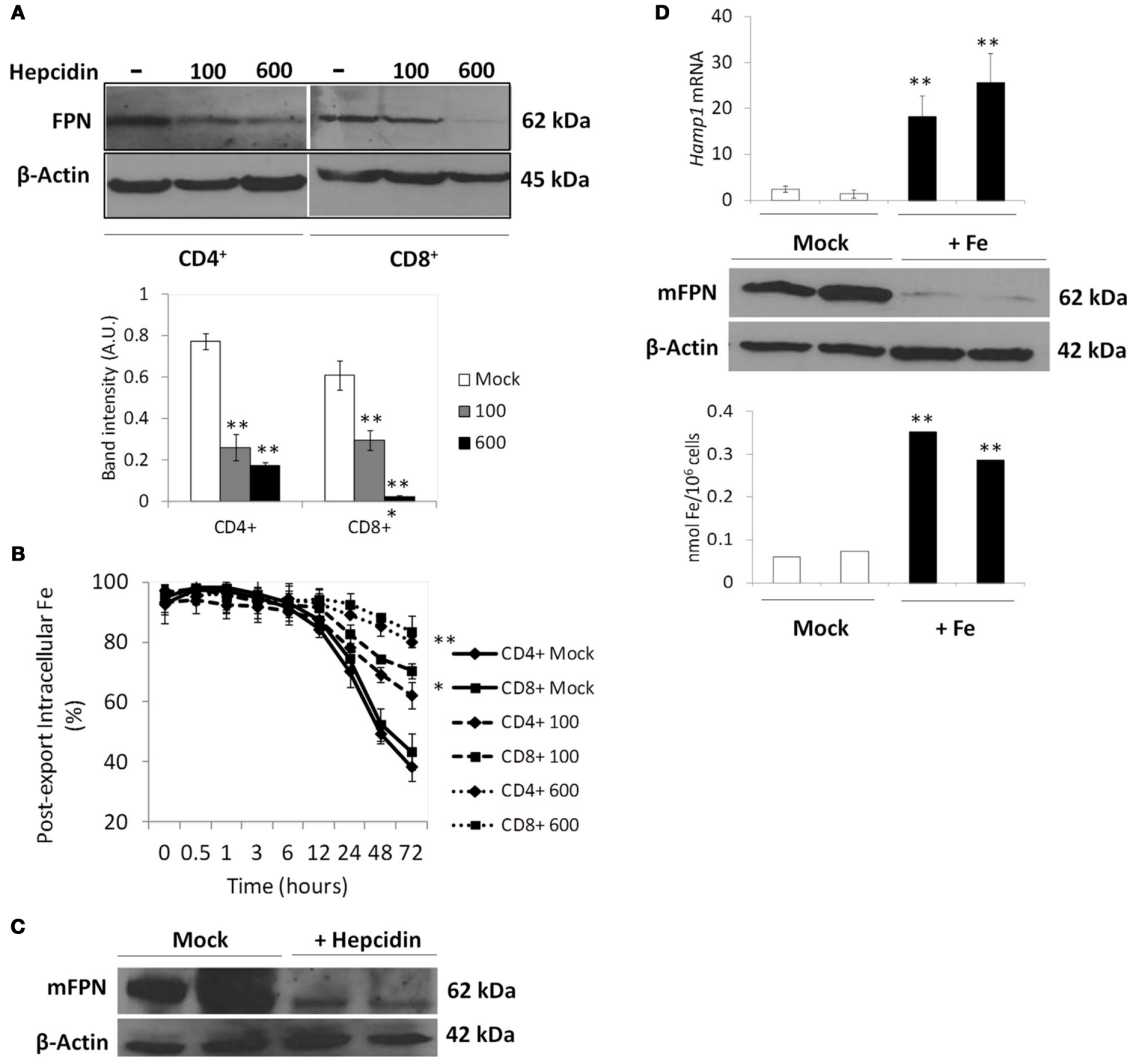
**Modulation of FPN1 expression and iron export by hepcidin in human and mouse T lymphocytes**. **(A)** Modulation of Ferroportin (FPN1) by hepcidin in human T lymphocytes. Western blot quantification of FPN1 in CD4^+^ and CD8^+^ T lymphocytes incubated with the indicated concentrations (ng/ml) of hepcidin for 24 h. β-actin expression was used as loading control. Images are representative of three experiments without significant inter-experimental variation. Bottom panel: Scanning densitometry of FPN1 Western blot. Data are normalized for β-actin levels and are expressed as arbitrary units (AU). ^**^P < 0.005 (One-Way ANOVA) between relative FPN1 levels in Mock- and hepcidin-supplemented conditions. **(B)** Modulation of iron retention by hepcidin. Each point represents the mean value ± 1 SD (*n* = 3) of intracellular iron in human T lymphocytes incubated with 5 μM of ^55^Fe-citrate for 2 h and allowed to export iron in an NTBI-free medium, in the presence (100 or 600 ng/ml) or absence (Mock) of hepcidin, for up to 72 h. ^*^P < 0.05 and ^**^P < 0.005 (One-Way ANOVA) between the time-dependent distribution of iron levels in each experimental condition and the no-iron control, for each cell population. **(C,D)** *In vivo* modulation of mFpn1 by hepcidin and iron in mice T lymphocytes. **(C)** Western blot quantification of mFpn1 expression in T lymphocytes from C57Bl/6 mice injected with synthetic mouse hepcidin (+ Hepcidin) or with PBS (Mock). β-actin expression was used as loading control. Each lane represents a pool of CD3^+^ cells from 3 animals. **(D)** Upper panel: qRT-PCR quantification of *Hamp1* expression in livers (*n* = 3 per bar) of C57Bl/6 mice fed iron-rich (+Fe) or iron-sufficient (Mock) diets followed by injection of 50 μM ^55^Fe-citrate. Western blot quantification of Fpn1 expression (*middle panel*) and intracellular ^55^Fe quantification (*bottom panel*) in peripheral blood T lymphocytes collected from the same animals. In middle and bottom panels data from each lane represents a pool of T lymphocytes from 6 animals. ^**^P < 0.005 (Student's *t*-test).

Besides the IRE/IRP translational regulation, FPN1 expression is also regulated post-translationally by the hormone hepcidin, which is secreted mainly by the liver in response to several stimuli, including iron overload (Nemeth et al., 2004). Since the characterization of iron export above was performed in a hepcidin-free medium, which does not correspond to most *in vivo* situations in which NTBI is present, we re-analyzed iron export by T lymphocytes, using the same experimental setup, in the presence of human synthetic hepcidin at doses present in human serum in normal and in iron-overload conditions (Ganz et al., 2008). Exogenous hepcidin caused a dose-dependent decrease in FPN1 levels in both T lymphocyte populations (Figure 4A) together with a significant dose-dependent increase in iron retention over 72 h (Figure 4B). To control for possible hepcidin-mediated changes in NTBI uptake that could explain the iron-retention results we measured iron uptake in T lymphocytes incubated with 5 μM of ^55^Fe-citrate for 30 min, in the presence of human hepcidin. No significant differences were found between hepcidin-supplemented and hepcidin-free conditions (Supplementary Figure 3), which leads us to ascribe the effect of hepcidin exclusively on iron export.

The previous results show that iron export by T lymphocytes is virtually totally abolished following exposure to hepcidin levels typically present in iron overload and inflammation contexts (600 ng/ml), suggesting that systemic hepcidin levels may significantly modify the amount of iron which is released by T lymphocytes back into the circulation. To test this hypothesis, C57Bl/6 mice were intravenously injected with mouse synthetic hepcidin, or PBS, for 3 consecutive days and Fpn1 expression quantified in peripheral blood CD3^+^ cells. Confirming the *ex vivo* results, hepcidin injections caused a marked decrease of Fpn1 in mouse peripheral blood T lymphocytes (Figure 4C). To further explore the involvement of systemic hepcidin in the control of iron export by T lymphocytes, C57Bl/6 mice were fed an iron-rich diet for 2 weeks, in order to saturate transferrin and induce an increase in systemic hepcidin levels. Controls were fed a diet previously shown to be iron-sufficient (Ramos et al., 2011). As expected, dietary iron induced a significant increase in serum iron parameters, including transferrin saturation, which increased from 53 ± 8% in control animals to 87 ± 4% in mice on iron-rich diet (Table 1). Two weeks later, all mice were intravenously injected with 100 μl of 50 μM ^55^Fe-citrate (50:100), to achieve a systemic ^55^Fe-citrate concentration of 5 μM. Analysis of the animals 48 h after injection showed, as expected, a significant increase (about 20-fold) of liver *Hamp1* mRNA levels in iron-loaded mice (Figure 4D). Most importantly, peripheral blood CD3^+^ cells in iron-overloaded mice had a marked reduction in Fpn1 expression and significantly higher intracellular ^55^Fe levels. Altogether, these results demonstrate, *in vivo*, the role of hepcidin in the control of NTBI retention vs. export by peripheral blood T lymphocytes.

**Table 1:** **Serum iron-related parameters in mice fed iron-sufficient and iron-enriched diets**.

**Blood iron-related parameters/Experimental condition**	**UIBC (μ g/dl)**	**TIBC (μ g/dl)**	**Serum Fe (μ g/dl)**	**Tf saturation (%)**
Fe-sufficient diet	146.25 ± 53.5	303.75 ± 60.2	158 ± 16	53 ± 8
Fe-enriched diet	46.25 ± 15.2	358.25 ± 11.1	312 ± 6	87 ± 4

*UIBC, unsaturated iron-binding capacity; TIBC, total iron-binding capacity; Tf, transferrin*.

#### Systemic impact of NTBI uptake by T lymphocytes

**Figure 5:**
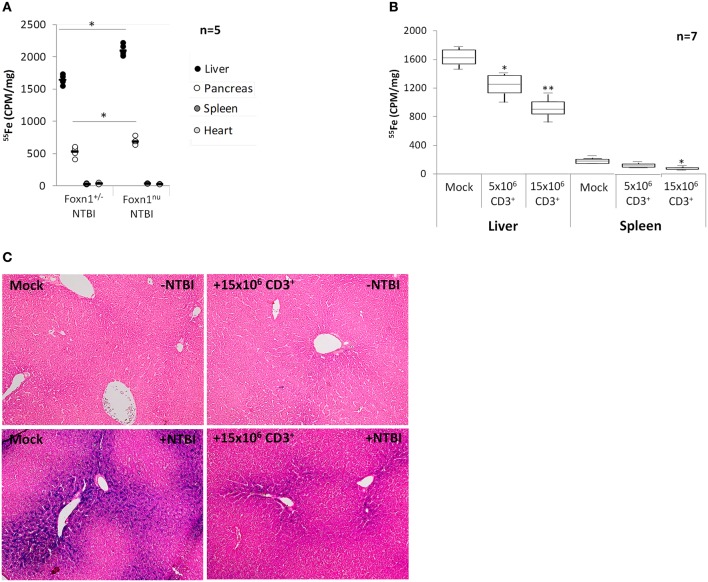
**NTBI accumulation in T lymphocyte-deficient and T lymphocyte-reconstituted mice. (A)** ^55^Fe levels in livers, pancreas, spleens, and hearts of *Foxn*^+/−^ and *Foxn1^nu^* mice fed an iron-rich diet and injected with 50 μM ^55^Fe-citrate. Each dot represents an animal. ^*^P < 0.05, *Foxn1^nu^* vs. the corresponding tissue of *Foxn*^+/−^ mice; Students' *t*-test. **(B)** ^55^Fe levels in livers and spleen of *Foxn1^nu^* mice fed an iron-rich diet + injection of 50 μM ^55^Fe-citrate and reconstituted with 5 × 10^6^ or 15 × 10^6^ CD3^+^ T lymphocytes or injected with PBS (Mock). Data are depicted as lower quartile, median, and upper quartile (boxes) and minimum/maximum ranges (whiskers; ^*^P < 0.05, ^**^P < 0.005 control vs. reconstituted animals; Students' *t*-test). **(C)** Perls' Prussian blue staining of iron (blue) in livers of *Foxn1^nu^* mice + iron-sufficient diet (upper left panel), *Foxn1^nu^* mice + iron-sufficient diet reconstituted with 15 × 10^6^ T lymphocytes (upper right panel), *Foxn1^nu^* mice + iron-rich diet (bottom left panel) and *Foxn1^nu^* mice + iron-rich diet reconstituted with 15 × 10^6^ T lymphocytes (bottom right panel). Magnification = 100x.

**Figure 6:**
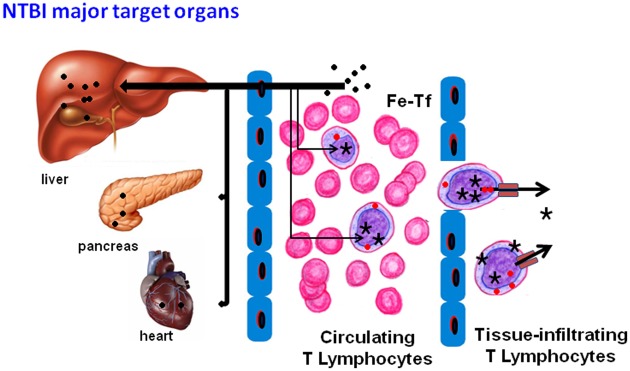
**Proposed model of NTBI storage and further utilization for cell growth**. When in contact with hepatocytes, NTBI (●) is rapidly taken up and stored, this being classically accepted as the most effective mechanism of NTBI clearance from the blood. NTBI in the blood stream is taken up by T lymphocytes, where it integrates the intracellular labile iron pool (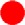) and the ferritin stores (★). These cells, and possibly others, constitute the “circulating” iron storage compartment. When the circulating and liver NTBI clearance capacities are exceeded, other cell types, such as pancreatic beta cells or cardiomyocytes, also take up NTBI, with iron accumulation in the respective organs (pancreas, heart). Besides constituting a first line “safe” deposit of iron, T lymphocytes (and possibly other circulating cells) could also contribute to iron distribution and delivery to other cells and tissues, either in normal physiological (homeostatic erythroid/lymphoid proliferation) or pathological conditions (stress erythropoiesis, inflammation, tumor growth). T lymphocyte-derived iron uptake by target cells could occur as transferrin-bound iron (Fe-Tf) or as NTBI, which could be exported directly via ferroportin or, eventually, by ferritin secretion, as proposed earlier (Dörner et al., 1980).

The results above led us to hypothesize that NTBI storage by T lymphocytes may represent a modifier of accumulation of this iron form in target organs. To test this hypothesis, we first compared iron accumulation in target organs of T lymphocyte-deficient *Foxn1^nu^* and T lymphocyte-normal *Foxn1*^+/−^ mice fed an iron-rich diet in which traceable NTBI was generated by intravenous injection of ^55^Fe-citrate. We observed significantly higher ^55^Fe levels in the livers than in the other organs analyzed, confirming that the traceable iron in our experimental setup mostly incorporates the NTBI pool (Figure 5A). In accordance with previous results obtained with other lymphocyte-deficient models (de Sousa et al., 1994; Santos et al., 2000; Cardoso et al., 2002), there is a significant increase in iron accumulation in livers and pancreas of *Foxn1^nu^* mice, in comparison with *Foxn1*^+/−^ controls. No significant differences were observed for the spleen and heart. Next the specific involvement of T lymphocytes in this result was tested by reconstituting a group of *Foxn1^nu^* animals in iron-rich diets with 5 × 10^6^ or 15 × 10^6^ T lymphocytes isolated from the spleens of histocompatible *Foxn1*^+/−^ animals on iron-normal diets. In *Foxn1^nu^* animals reconstituted with 5 × 10^6^ T lymphocytes liver iron levels are approximately 20% lower than in non-reconstituted controls, a difference which increases to approximately 35% when 15 × 10^6^ T lymphocytes were used (Figure 5B). The same result, although with lower statistical significance, was observed for the spleen. Histological analysis of *Foxn1^nu^* liver sections showed no significant hemosiderin deposition in the livers of *Foxn1^nu^* or T lymphocyte-reconstituted *Foxn1^nu^* mice on iron-normal diets (Figure 5C), but high levels of stainable iron were detected in the liver parenchyma of *Foxn1^nu^* iron-loaded animals. Confirming the results obtained with ^55^Fe, reconstitution of the T lymphocyte pool with 15 × 10^6^ T lymphocytes reduced the levels of liver stainable iron. Changes in liver iron deposition in response to T lymphocyte reconstitution cannot be ascribed to alterations in intestinal iron absorption or iron retention induced by hepcidin, since no changes in hepcidin mRNA levels were observed in response to T lymphocyte transfer (Supplementary Figure 4). Altogether, these results demonstrate the physiological role of T lymphocytes as modifiers of NTBI deposition in target organs, which schematic representation is proposed in Figure 6.

## Discussion

The major players and mechanisms in systemic iron homeostasis classically consider four major compartments, involving particular cell types. The *functional* compartment (erythroid precursors or other proliferating cell pools), the *uptake* compartment (enterocytes), the *recycling* compartment (spleen macrophages), and the *storage* compartment (hepatocytes and macrophages). The described model, however, neglects the contribution of all remaining cell types which may contribute to the fine-tuning of the homeostasis of this essential yet dangerous element. In this work we demonstrate the capacity of peripheral blood circulating T lymphocytes to store iron acquired in the form of NTBI, which is the iron presentation associated with toxicity in iron-overload disorders. In addition, we describe how this capacity, modulated by the hepcidin-ferroportin axis, has important physiological consequences. By doing so, we introduce the concept of the “*circulating storage* compartment” and establish a role for T lymphocytes as important players in this new component of iron homeostasis.

The capacity to take up NTBI has been demonstrated for a variety of circulating blood cells, including reticulocytes and erythrocytes (Zhang et al., 2008; Prus and Fibach, 2011), lymphocytes (Arezes et al., 2013), monocytes, eosinophils, basophils, neutrophils, and platelets (Pinto et al., unpublished results and Hausmann et al., 1988). It is thus possible that all or at least some of these cell types play a role in the buffering of NTBI from target organs. That will depend not only on the quantitative NTBI retention capacity but also on the selectivity of each cell type for the NTBI species present in circulation in each particular context. Among these cells, T lymphocytes constitute a particularly interesting population and an obvious target for analysis. They are known for some time to be negatively associated with the severity of iron overload both in mice (de Sousa et al., 1994; Santos et al., 1996; Cardoso et al., 2002) and in HFE-HH human patients (Porto et al., 1997; Barton et al., 2005; Cruz et al., 2006), and we show now that they possess the necessary features to be acknowledged as important players in NTBI homeostasis:

### Fast and selective NTBI import

NTBI clearance from the circulation has been previously described to be a rapid and efficient procedure. Once in contact with the liver, NTBI is cleared with a half-life of <30 s, in comparison with approximately 50 min for TF-bound iron (Brissot et al., 1985). The same rapid pattern of NTBI uptake was previously described by us for T lymphocytes, although these cells show a lower capacity to accumulate NTBI than hepatocytes (Parkes et al., 1995; Arezes et al., 2013).

### Iron utilization/storage after exposure to NTBI

We show here an increase in the LIP of T lymphocytes exposed to NTBI. The nature and distribution of LIP in any cellular system remains essentially unknown. Our results with EDX analysis in T lymphocytes show a heterogeneous distribution of intracellular iron and the enrichment of iron-rich structures with nitrogen, oxygen, phosphorous and chlorine, suggesting the association of iron with compounds such as phosphate and proteins. This association may result in protection of the cell from oxidative damage mediated by labile iron, an hypothesis supported by previous studies showing the lack of NTBI-induced ROS in Jurkat cells that accumulate NTBI as phosphate nanoparticles (Jhurry et al., 2012, 2013).

Intracellular iron incorporated in the LIP fraction can either be utilized in iron-requiring processes or stored, mostly associated with ferritin. Evidence that T lymphocytes are equipped to integrate iron acquired as NTBI in the cell's metabolism is provided in our previous study showing an increase in activation-mediated proliferation in response to this iron form (Arezes et al., 2013). Evidence for iron storage is provided here by the observation of an NTBI-induced up-regulation of the ferritin levels in T lymphocytes, suggesting an increase in the iron-storage capacity of these cells in response to NTBI uptake. The few studies previously addressing the response of ferritin to NTBI in lymphocytes show disparate results and their interpretation is hindered by the use of distinct iron donors, most of them non-physiological, and of frequently non-physiological NTBI concentrations (Pelosi et al., 1986; Seligman et al., 1991; Djeha and Brock, 1992; Chitambar and Wereley, 2001). We believe that the use of ferric citrate as iron donor, the maintenance of citrate concentrations between the physiological interval of 60–140 μM (Lentner, 1984) and the use of iron concentrations in the range of those found commonly in iron overload situations (Evans et al., 2008) constitute the correct experimental setup and should become the standard procedure for future studies involving biological systems and NTBI.

### NTBI export in a regulated manner

We demonstrate that iron acquired as NTBI by T lymphocytes is exported via ferroportin in an IRP/IRE-regulated manner. *In vitro*, and in the absence of exogenous hepcidin, both T lymphocyte populations (CD4^+^ and CD8^+^) export approximately 3% per hour of intracellular Fe, which compares with what has been reported for monocytic/macrophagic cell lines—approximately 6% (Ludwiczek et al., 2003). However, these values might not represent the *in vivo* iron export of these cells, as the demonstration of the modulation of T lymphocyte ferroportin, and concomitant Fe export, by circulating hepcidin, places the effective iron export of T lymphocytes, and thus their NTBI storage capacity, under the systemic control. This finding, together with a previous study reporting the expression of ferroportin by erythroblasts and its modulation by hepcidin (Zhang et al., 2011), places this protein in an even more central place in iron homeostasis and highlights the need to consider the contribution of other “non-classical” players in pathologies associated with inappropriate expression of hepcidin. Nevertheless, these results do not preclude the existence of alternative iron export pathways, such as iron exported in association with ferritin, a mechanism first proposed for T lymphocytes (Dörner et al., 1980) and later described for macrophages (Cohen et al., 2010) and hepatocytes (Nemeth, pers. comm.).

Our results show that CD4^+^ and CD8^+^ T lymphocytes do not differ significantly in the export/retention of iron acquired as NTBI. Previous results showing iron overload in mice deficient in CD8^+^ T lymphocytes (de Sousa et al.1994x; Santos et al., 1996; Cardoso et al., 2002) and the inverse correlation recurrently observed between CD8^+^ T lymphocyte numbers and the severity of iron overload in HFE-HH human patients (Porto et al., 1997; Barton et al., 2005; Cruz et al., 2006) might thus need to be reinterpreted not as an indication that CD8^+^ T cells play a unique role in iron homeostasis but instead that the lack or a reduction in number of these cells affects the fine-tuning of iron homeostasis, namely NTBI distribution. This conclusion is supported by previous results showing higher severity of iron overload in β_2_m^−^Rag1^−^ mice—deficient in CD4^+^ and CD8^+^ T lymphocytes and B lymphocytes—than in β_2_m^−^ animals—deficient in CD8^+^ T lymphocytes (Santos et al., 2000). The lack of differences in NTBI retention between CD4^+^ and CD8^+^ cells is also in agreement with our previous findings of similar NTBI uptake by the two cell types (Arezes et al., 2013). In that study we did not find evidence for the involvement of neither DMT1 nor ZIP14, the most likely NTBI transporter candidates (Trinder et al., 2000; Liuzzi et al., 2006), leaving the door open for the identification of a still elusive transport mechanism.

Here we demonstrate the ability of transferred T lymphocytes to retain circulating NTBI and thus reduce its accumulation in the liver and spleen. This result cannot be ascribed to hepcidin-mediated changes in iron metabolism induced by T lymphocyte transfer, since (1) no significant differences in liver hepcidin expression were found in response to transference alone and (2) no visible changes in liver iron deposition were observed between non-transferred and transferred animals on an iron-sufficient diet. This is in agreement with a previous report showing the absence of alterations in iron homeostasis in NOD/SCID mice transferred with syngeneic T lymphocytes (Bair et al., 2009). The lack of increased iron deposition in the livers of T lymphocyte-depleted animals on an iron sufficient diet also illustrates one important conclusion from our results which is that the absence of T lymphocytes *is not the cause* of iron overload and NTBI accumulation but instead that, by acting as a first line of retention, T lymphocytes *are modifiers* of NTBI accumulation when this iron form is present in the blood circulation.

At the present time we cannot exclude the possibility that other mechanisms besides NTBI storage could, at least in part, underlie the observed reduction in organ iron deposition. An alternative explanation is the synthesis of cytokines or other molecules by T lymphocytes upon contact with iron, which could in turn impact, directly or indirectly, on the handling of iron by other tissue/cell types, as previously reported (Ten Elshof et al., 1999; Meyer et al., 2002; Sharma et al., 2009).

The capacity of T lymphocytes to store and release NTBI may have implications beyond their capacity to modify systemic iron overload. It is reasonable to consider that at the local tissue level they could interfere with the availability of iron in the extracellular milieu (as proposed in Figure 6) and thus influence the growth rate of adjacent cells, playing a role in normal cell/tumor growth and tissue remodeling, such as already demonstrated for polarized M2 macrophages (Recalcati et al., 2012). This report does not address the impact of NTBI uptake in the *in vivo* T lymphocyte metabolism besides iron storage and export. Although in our experimental conditions T lymphocytes retained their viability and proliferation potential, at this point we cannot exclude the possibility that accumulation of iron from NTBI may interfere with other normal T lymphocyte functions and immune surveillance. Previous studies have shown that lymphocytes from iron-overloaded animals have a reduced capacity to generate allo-specific cytotoxic responses (Good et al., 1987) and that ferric salts can alter a variety of lymphocyte functions *in vitro* (Nishiya et al., 1980; Brock, 1981; Bryan et al., 1981, 1986; van Asbeck et al., 1982; Bryan and Leech, 1983). In addition, specific abnormalities in CD8^+^ T lymphocyte functions have been described in HFE-HH patients, including defective lymphocyte-specific protein tyrosine kinase (p56lck) activity, decreased cytotoxic activity, a decreased number of CD8^+^ T cells expressing the co-stimulatory molecule CD28, but also an increased number of CD8^+^ T cells lacking CD28, and an abnormally high percentage of HLA-DR-positive activated T cells (Arosa et al., 1994, 1997; Arosa, 2002).

In conclusion, this work demonstrates that T lymphocytes are important components of a circulating NTBI storage compartment and show its physiological relevance as modifiers of tissue iron overload. On a broader scope, it provides a mechanistic support for the possibility of circulating T lymphocytes acting *in vivo* as a key component in systemic iron homeostasis, by playing a role in the surveillance of iron toxicity and of its possible use by pathogens and tumor cells, as first postulated 32 years ago (de Sousa, 1981).

## Author contributions

Jorge P. Pinto conceptualized the idea, designed and performed the research, analyzed data, and wrote the paper; João Arezes performed the research, analyzed data and wrote the paper; Vera Dias, Susana Oliveira, Inês Vieira, Mónica Costa, Matthijn Vos, Anna Carlsson, and Yuri Rikers performed the research; Maria Rangel analyzed data and wrote the paper; Graça Porto contributed with vital analytical tools, contributed to design of the research, analyzed data and wrote the paper.

### Conflict of interest statement

The authors declare that the research was conducted in the absence of any commercial or financial relationships that could be construed as a potential conflict of interest.

## Supplementary material

Supplementary Figure 1 **Assessment of the purity of the plasma membrane fraction from human CD3^+^ cells**. Membrane (M; *n* = 1), cytoplasmic (C; *n* = 3), and total (T; *n* = 1) protein fractions from human CD3^+^ cells were blotted and the membrane incubated with the plasma membrane marker anti-alpha 1 Na/K ATPase antibody.

Supplementary Figure 2 **Effect of NTBI on *FPN1* mRNA expression in human peripheral blood T lymphocytes**. qRT-PCR analysis of *FPN1* mRNA expression in human peripheral blood CD4^+^ and CD8^+^ T lymphocytes incubated with 5 μM Fe-citrate or PBS for up to 6 h. Data are depicted as fold change relative to *FPN1* mRNA levels at 0 h for each cell population. Experiments were performed three times with 2 replicates per experiment. Each bar represents the mean ± 1 SD.

Supplementary Figure 3 **Effect of hepcidin on Fe-citrate uptake by T lymphocytes**. Intracellular ^55^Fe levels in CD4^+^ and CD8^+^ T-lymphocytes incubated with 5 μM of ^55^Fe-citrate, for 30 min, in the presence of 100 or 600 ng/ml of human synthetic hepcidin. Mock controls were incubated without hepcidin. Experiments were performed three times with 3 replicates per experiment. Each point represents the mean ± 1 SD.

Supplementary Figure 4 **Effect of T lymphocyte reconstitution on *Hamp1* mRNA expression on *Foxn1^nu^* mice**. qRT-PCR quantification of *Hamp1* mRNA expression on livers of *Foxn1^nu^* mice on iron-rich (+NTBI) or iron-sufficient (no NTBI) diets I.V. injected with 15 × 10^6^ CD3^+^ T lymphocytes or with PBS (Mock). Data are depicted as fold change relative to *FPN1* mRNA levels of Mock animals on iron-sufficient diet. Each bar represents the mean ± 1 SD of the values obtained for three animals. Statistical analysis was performed using One-Way ANOVA.
