# Supplementary figures and images for: Physiological implications of NTBI uptake by T lymphocytes
Source: Front Pharmacol. 2014 Feb 26;5:24. doi: 10.3389/fphar.2014.00024 (PMC3935319; doi:10.3389/fphar.2014.00024)

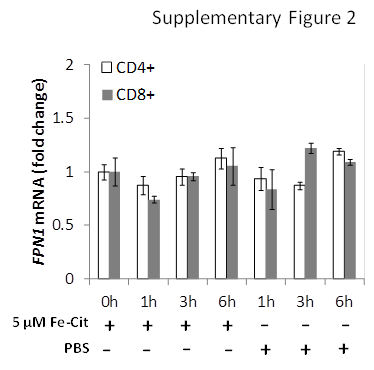

Supplement: Supplementary Figure 1 — Assessment of the purity of the plasma membrane fraction from human CD3+ cells. Membrane (M; n = 1), cytoplasmic (C; n = 3), and total (T; n = 1) protein fractions from human CD3+ cells were blotted and the membrane incubated with the plasma membrane marker anti-alpha 1 Na/K ATPase antibody. [file Presentation1.ZIP › 79471_Porto_Suppl_Figure_2.TIF]

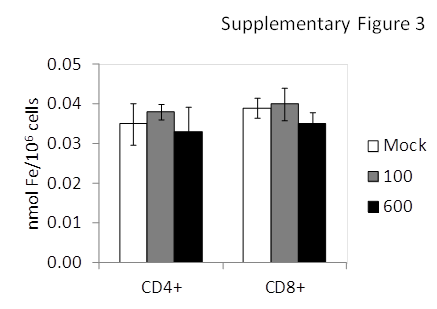

Supplement: Supplementary Figure 1 — Assessment of the purity of the plasma membrane fraction from human CD3+ cells. Membrane (M; n = 1), cytoplasmic (C; n = 3), and total (T; n = 1) protein fractions from human CD3+ cells were blotted and the membrane incubated with the plasma membrane marker anti-alpha 1 Na/K ATPase antibody. [file Presentation1.ZIP › 79471_Porto_Suppl_Figure_3.TIF]

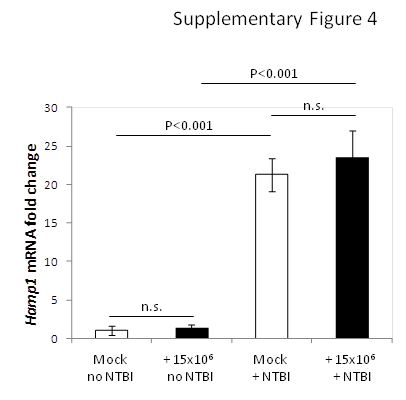

Supplement: Supplementary Figure 1 — Assessment of the purity of the plasma membrane fraction from human CD3+ cells. Membrane (M; n = 1), cytoplasmic (C; n = 3), and total (T; n = 1) protein fractions from human CD3+ cells were blotted and the membrane incubated with the plasma membrane marker anti-alpha 1 Na/K ATPase antibody. [file Presentation1.ZIP › 79471_Porto_Suppl_Figure_4.TIF]

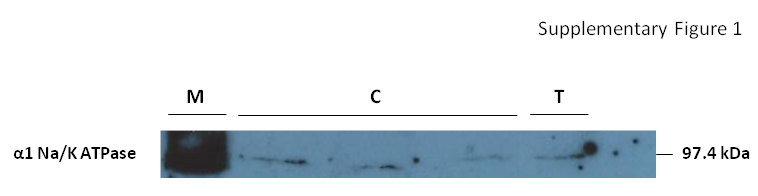

Supplement: Supplementary Figure 1 — Assessment of the purity of the plasma membrane fraction from human CD3+ cells. Membrane (M; n = 1), cytoplasmic (C; n = 3), and total (T; n = 1) protein fractions from human CD3+ cells were blotted and the membrane incubated with the plasma membrane marker anti-alpha 1 Na/K ATPase antibody. [file Presentation1.ZIP › 79471_Porto_Suppl_Figure_1.TIF]
